# Trace Metal Element Analysis in Some Seafood in the Coastal Zone of the Red River (Ba Lat Estuary, Vietnam) by Green Sample Preparation and Inductively Coupled Plasma-Mass Spectrometry (ICP-MS)

**DOI:** 10.1155/2021/6649362

**Published:** 2021-03-04

**Authors:** Nhu Da Le, Thi Thu Ha Hoang, Vu Phong Phung, Thi Lien Nguyen, Thi Thuy Duong, Le Minh Dinh, Thi Mai Huong Pham, Thi Xuan Binh Phung, Tien Dat Nguyen, Thanh Nghi Duong, Thi My Hanh Le, Phuong Thu Le, Thi Phuong Quynh Le

**Affiliations:** ^1^Institute of Natural Product Chemistry (INPC), Vietnam Academy of Science and Technology, 18 Hoang Quoc Viet, Cau Giay, Hanoi, Vietnam; ^2^Graduate University of Science and Technology, Vietnam Academy of Science and Technology, 18 Hoang Quoc Viet, Cau Giay, Hanoi, Vietnam; ^3^Institute for Technology of Radioactive and Rare Elements, 48 Lang Ha Str., Dong Da, Hanoi, Vietnam; ^4^Institute of Environmental Technology, Vietnam Academy of Science and Technology, 18 Hoang Quoc Viet, Cau Giay, Hanoi, Vietnam; ^5^Hanoi University of Industry, 298 Cau Dien, Bac Tu Liem, Hanoi, Vietnam; ^6^Electric Power University, 235 Hoang Quoc Viet, Bac Tu Liem, Hanoi, Vietnam; ^7^Center for Research and Technology Transfer, Vietnam Academy of Science and Technology, 18 Hoang Quoc Viet, Cau Giay, Hanoi, Vietnam; ^8^Institute of Marine Environment and Natural Resources, Vietnam Academy of Science and Technology, Hai Phong, Vietnam; ^9^Institute of Tropical Technology, Vietnam Academy of Science and Technology, 18 Hoang Quoc Viet, Cau Giay, Hanoi, Vietnam; ^10^University of Science and Technology of Hanoi, Vietnam Academy of Science and Technology, 18 Hoang Quoc Viet Road, Cau Giay, Hanoi, Vietnam

## Abstract

Fisheries and aquaculture production in the coastal zone of Vietnam contribute significantly to the national economy. However, seafood quality and safety, especially in terms of metal contents, are of increasing concern, for both domestic and international markets. This paper presents the results of an investigation in some trace metal elements (iron (Fe), zinc (Zn), manganese (Mn), copper (Cu), arsenic (As), cadmium (Cd), and mercury (Hg)) concentrations in some fishes, crustaceans, and molluscs in the coastal zone of the Red River (in the Ba Lat estuary in Thai Binh and Nam Dinh provinces) during four sampling campaigns in 2020. All samples were treated by a green sample preparation using microwave digestion and then analyzed by inductively coupled plasma-mass spectrometry (ICP-MS). The results showed that the trace metal element concentrations in fish, crustacean, and mollusc samples decreased in the following order: Fe > Zn > Mn > Cu > As > Cd ∼ Hg. In more details, the ranges of trace metal elements in seafood samples were 13.13–202.73; 7.63–82.71; 0.48–22.73; 0.72–15.58; 0.18–5.12; 0.001–1.114; and 0.001–0.923 mg·kg^−1^ for Fe, Zn, Mn, Cu, As, Cd, and Hg, respectively. The research results contribute to the dataset of the seafood (both fishery and aquacultural seafood) quality in the Red River coastal zone. Although the mean values of different trace metal elements observed in this study were lower than the allowed values of Vietnam's or European's threshold for food safety, some high concentrations were detected. The survey results suggest the need to expand the monitoring scope (frequency of monitoring, number of samples, and observed variables) for obtaining a fully comprehensive assessment of seafood quality in this region. Our results also indicate that it is necessary to manage water quality in coastal areas, especially where aquaculture activities are carried out.

## 1. Introduction

Seafood is classified as nutritious and protein-rich foods, providing essential and trace elements (Zn, Fe, Cu,…) as nutrients for human health. However, some seafood has the ability to absorb and accumulate trace metal elements in their bodies and which then affect human health when people consume seafood [1]. In parallel with the increasing demand of food safety and food consumption, trace metal element accumulation in seafood, especially in major seafood products, is particularly of great concern. Trace metal elements accumulated in different organisms are closely related to their difference in biokinetics. Wang and Guangyuan [2] review the concentrations of several major metal contaminants in bivalve molluscs collected from different regions of the world and found that oysters are the hyperaccumulators of Cu and Zn, whereas scallops are the hyperaccumulator of Cd. Thus, many species are used as environmental indicators to alert and assess water pollution. For example, oysters are usually used as a bioindicator of Cu and Zn contamination [3, 4].

Several analytical methods are used for determining trace metal element contents in seafood. Most studies use atomic absorption spectrometry (AAS) for analyzing trace metal elements in seafood, such as the determination of Cr, Mn, Cu, Zn, As, Cd, Hg, and Fe in fish and crustacean samples in Bangladesh [5] or the study of Cd, Pb, and Hg in fish and seafood products in Bosnia and Herzegonia [6] or Fe, Mn, Cu, Zn, Pb, Ni, Cd, and Co in molluscan shells in the Gulf of Aqaba and Red Sea coasts, Egypt [7]. Another method, inductively coupled plasma-mass spectrometry (ICP-MS), is also utilized for analyzing different trace metal elements in edible fish from Atlantic Coast of Muanda, Congo [8], or in shrimp and shellfish in South Korea [9]. The ICP-MS method is largely used because of its multiple advantages including the low detection limits (0.01 to 0.1 *μ*g·L^−1^ for many elements), simple specimen preparation, high throughput, and the ability to measure multielements simultaneously.

The coastal zone of the Ba Lat estuary (Red River) in Nam Dinh and Thai Binh provinces in North Vietnam is targeted to become a nationally and internationally important economic zone by the Vietnamese government. In this region, fisheries and aquaculture play an important role in the provincial economy through their role as a source of food, nutrition, income, and livelihoods for local inhabitants. Like other regions in the world, while the quantity of fisheries has increased relatively little, aquaculture in this region has been responsible for the impressive growth in the food supply for human consumption since 1995 [10].

Water and sediment quality in the coastal zone is one of the important factors that affect seafood quality in both fisheries and aquaculture sectors. Recently, some observation results of environmental quality in Thai Binh and Nam Dinh provinces showed that water and sediment environment had signs of contamination, including trace metal elements. Le Thi Lai et al. [11] reported that water channels in handicraft villages in Nam Dinh Province are loaded with trace metal elements (Zn, Pb, Cu, Cd, Cr, and Fe), exceeding the limits by up to 50 times. Some trace metal element (Fe, Zn, and Cu) concentrations exceeded the permissible limits for coastal seawater quality QCVN 10-MT:2015/BTNMT [12, 13]. At the Ba Lat estuary, at some monitoring time, Fe concentrations in the water were from 2.2 to 9.7 times higher than the allowed value QCVN 10-MT: 2015/BTNMT [12] whereas Zn concentrations were from 1.28 to 5.12 times higher than the standard limits [14]. High contents of trace metal elements (Cu, Zn, Pb, As, Cd, and Hg) in the coastal sediment of North Vietnam, including Thai Binh and Nam Dinh provinces, were also observed in different studies [15, 16]. This may affect the life and quality of the fishery and aquacultural seafood in this area. Therefore, the assessment of trace metal element contents in fishery and aquacultural seafood is very important in the region. However, the study on trace metal element bioaccumulation in seafood in this region is still limited.

In the present study, we aim to apply a method basing on the digestion by microwave and the analysis by inductively coupled plasma-mass spectrometry (ICP-MS) to evaluate seven trace metal elements (Fe, Zn, Mn, As, Cu, Cd, and Hg) in three kinds of fishery and aquacultural seafood (fish, crustacean, and mollusc samples) which are widely available and major seafood products in the coastal area of the Red River in Thai Binh and Nam Dinh provinces. The research results contribute to the dataset construction of the seafood quality in this region and provide a scientific basis for the planning management for better protection and sustainable development of aquaculture in the study area.

## 2. Study Site and Methodology

### 2.1. Study Site

Thai Binh Province covers a surface area of 1,586 km^2^ with a total population of 1.86 10^6^ inhabitants in 2019 whereas Nam Dinh Province has 1,668 km^2^ with 1.78 10^6^ inhabitants [10].

Meteorological and hydrological characteristics: the climate in this region is characterized by two distinct seasons: the rainy season (from May to October) often accounts for 85–90% of the total annual rainfall (1700–1800 mm·yr^−1^) and the dry season (from November to next April). The monthly average air temperature ranges from 14 to 27°C. In this region, the tidal regime is diurnal with an average tidal of 1.6 to 1.7 m. The highest tidal is 3.31 m, and the smallest is 0.11 m [10, 17].

Fishery and seafood aquaculture have developed in both Thai Binh and Nam Dinh provinces. For example, Nam Dinh Province has a brackish and marine surface area of 15,200 hectares for aquaculture and a total exploited seafood fishery of about 149,639 tons in 2018. Similarly, Thai Binh Province has a marine and brackish surface area of 15,200 hectares for aquaculture (of which 3,000 ha for clams) and a total production of about 229,143 tons (in 2018) [10]. More than 25% of farmers living along coastal areas seek their livelihood from coastal fishery and aquaculture activities. Note that these two provinces, especially some coastal districts such as Giao Thuy, Thai Thuy, and Tien Hai, are considered as the largest areas of clam (*Meretrix* sp.) production in the North and Northcentral coastal region of Vietnam [18, 19]. Agriculture mainly rice culture also contributes an important proportion to the provincial economy in this region. Most farmers have been traditionally living on food crop production and animal breeding. Tourism activities are also developing in this region due to the presence of a mangrove ecosystem and a natural beach. Small and medium industrial factories or traditional production villages for food/seafood processing, gas exploitation, and crowded circulation at seaports are also present.

Along the coastal line of these two provinces, wastewater effluents originate from inland sources including domestic villages, rice fields, factories, and inland aquaculture farms. In addition, agricultural soils in this region are contaminated by several metal element (e.g., As and Cd) crust [17]. The coastal region receives also the considerable pollutant fluxes of the Red River, which discharges directly through the Ba Lat mouth [20, 21]. Hoai et al. [15] emphasized that metal contents in sediments in the coastal zone of North Vietnam have increased with time, as a consequence of the socioeconomic development in this region.

### 2.2. Sample Collection

**Figure 1:**
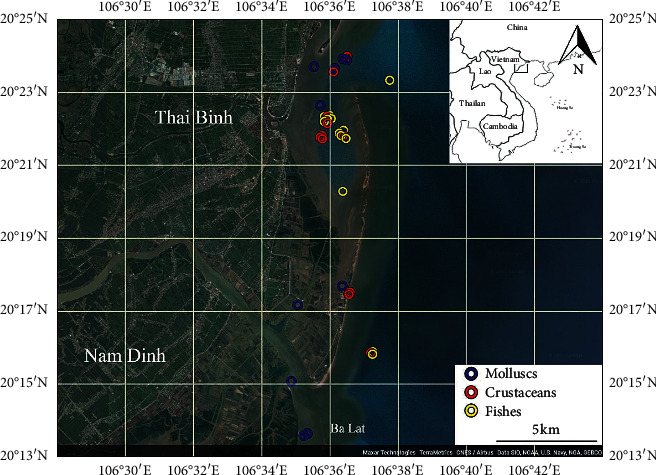
The Ba Lat estuary of the Red River, the coastal zone of the Thai Binh and Nam Dinh provinces.

A total of 35 seafood samples were collected from cultivated aquaculture farms (for mollusc samples) and natural coastal zone (fish and crustacean samples) of Nam Dinh and Thai Binh provinces: 13 different fish samples, 12 different crustacean samples, and 10 different mollusc samples (Table 1; Figure 1). The selected organisms are widely available as major seafood products in this region. The samples were collected in four sampling campaigns in 2020 in which two sampling campaigns were organized in the dry season (January and March) and another two in the rainy season (July and August) along the coastal zone of the Thai Binh and Nam Dinh provinces. Samples were collected by the Vietnam standard method TCVN 5276-90 for aquatic products, sampling and preparation of the sample. The samples collected in aquaculture coastal zone were clams (*Meretrix* sp.) cultured in Giao Thuy and Tien Hai districts whereas the samples in natural zone were different wild species of fishes and crustaceans which were randomly collected along the coastal zone of the Thai Binh and Nam Dinh (Table 1; Figure 1).

### 2.3. Sample Treatment and Analysis

Sample storage: the samples were thoroughly washed with ultrapure water and then stored in plastic bags in an ice bucket. In the laboratory, the samples were preserved at −20°C before analysis.

Sample treatment: the samples were dissected and the selected dorsal muscle tissues were lyophilized for 48 hours (Cryotec). The mass of each sample before and after freeze-drying was weighed. The dried sample was then finely ground into a powder.

In order to determine the metal concentration, 0.250 to 0.500 g each dried sample was weighed and digested with 1.5 mL of ultrapure HNO_3_ 65% (Merck, Germany) and 2 mL of ultrapure H_2_O_2_ 30% (Merck, Germany) in a Teflon bomb to solubilize the metal into the ionic form. The vessels were kept at room temperature for about 1 hour before heating.

A tray of a total of 12 samples was placed on a rotary table in a microwave digestion system (Q1716, Questron, USA). As compared to the open digestion methods, the biggest benefit of microwave digestion is time-saving, lower acid consumption, preventing the loss of volatile elements, and especially avoiding exposure of analyst to corrosive acid fumes. In this study, the microwave power (800 W) was set to be 30% for 3 min (step 1), then at 60% for 6 min (step 2), and at 30% for 1 min (step 3).

After being cooled to room temperature (25°C), the content in each Teflon vessel was transferred quantitatively into 25 ml volumetric flask, then filled up to volume with ultrapure Milli-Q water (18 MΩ). The samples were filtered through 0.45 *μ*m membrane (Whatman, Merck, Germany) before the measurement.

Trace metal elements were analyzed by ICP-MS analyzer (7700x, Agilent, USA) and quantified under specific wavelength conditions with the corresponding dilutions using ultrapure Milli-Q water, in order to be into the quantification range of each compound. Standards were simultaneously analyzed with experimental samples. Instrument deviation was checked at the beginning and the end of each measured trace element. In order to determine the background values of each species during the study period, blank samples (i.e., unexposed control filters) were routinely analyzed. Then, the real concentration of each species was calculated by subtracting the blank values from the results of the chemical analysis conducted on the exposed filters. Blank and replicated sample analyses were carried out in the same way.

All glassware, Teflon vessels were decontaminated by immersion in 10% v/v HNO_3_ (ultrapure, Merck, Germany) for at least 24 h and rinsed with ultrapure Milli-Q water.

The operating conditions of ICP-MS were optimized by using a mass standard solution to obtain the ratios of oxide ions (Ce^+^O/Ce) and doubly positive charged ions (Ce^2+^/Ce^+^) at the values of about 1.0 and 2.5%, respectively (Table 2).

The LOD and LOQ are calculated by the following equation:

(1) $$\begin{array}{c} \text{LOD}=\frac{3S\cdot C_{\text{STD}}}{I_{\text{std}}-I_{\text{blank}}}, \end{array}$$

(2) $$\begin{array}{c} \text{LOQ}=\frac{10S\cdot C_{\text{STD}}}{I_{\text{std}}-I_{\text{blank}}}, \end{array}$$

where 3 is a confidence factor; *S* is the standard deviation from 10 measurements of 10 blank samples; *C*_STD_ is the concentration of the standard sample (*μ*g·L^−1^); *I*_STD_ is the raw intensity of the standard sample (cps); and *I*_blank_ is the raw intensity of the blank sample (cps).

The limit of detection calculated by equation (1) for Fe, Zn, Mn, As, Cu, Cd, and Hg was 0.15, 1.17, 0.02, 0.09, 0.07, 0.01, and 0.27 *μ*g·L^−1^, respectively (Table 3).

The target analytes were not detectable in the blank. The quality control (QC) samples of 10 *μ*g·L^−1^ were diluted from standard stock solution (Inorganic Venture, USA) to evaluate the stability of the ICP-MS system. The percent recovery of the quality control sample (QCS) was within 90–102%, which was acceptable for the levels of the target analytes, according to the Manual on Policies and Procedure [22], indicating the absence of a significant analytical bias.

All samples were measured three times. The % RSD value was below 10% (Association of Official Analytical Chemists AOAC) was accepted.

### 2.4. Statistical Analysis

To detect the correlation between seven trace metal elements of 35 observed samples, the statistical software XLSTAT [23] was used to calculate the Pearson correlation coefficients. Principal component analysis (PCA) was then used to identify representative variables for each group (fish, crustacean and mollusc samples).

Student's *t*-test was used to test the difference of variable values between two seasons (wet and dry). Probabilities (*p*) were determined, and a *p* value of <0.05 was considered to be significant.

## 3. Results and Discussion

### 3.1. Trace Metal Element Concentrations in Fish, Crustacean, and Mollusc Samples

**Figure 2:**
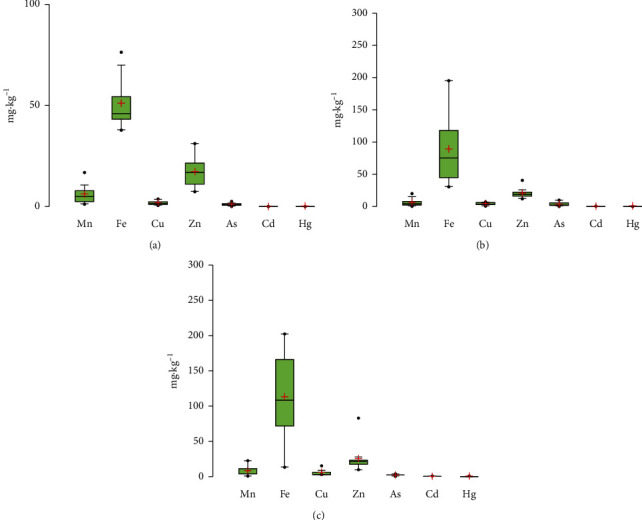
Trace metal element contents in (a) Fishes, (b) Crustaceans, and (c) Moluscs in the Red River coastal zone.

Fe is an essential nutrient for many organisms, especially for humans. Fe deficiency can lead to anemia and impaired intellectual development; however, higher Fe concentrations (e.g, 60 mg·kg^−1^ for one serving) can cause multiorgan failure, coma, seizures, and even death [24, 25]. In this study, the average contents of Fe were quite high, 51.23 ± 13.81 mg·kg^−1^ for fish, 89.92 ± 58.65 mg·kg^−1^ for crustaceans, and 113.14 ± 65.91 mg·kg^−1^ for mollusc samples (Table 4, Figure 2). Note that there is no available regulation for the permissible value of Fe content in fishery seafood.

Mn is an essential element as an antioxidant, for blood sugar regulation and bone growth [28]; however, high Mn contents can affect human health causing a Parkinson-like syndrome [29]. The mean contents of Mn were 5.99 ± 4.72 mg·kg^−1^ for fish, 6.37 ± 6.04 mg·kg^−1^ for crustaceans, and 8.24 ± 8.26 mg·kg^−1^ for mollusc samples (Table 4, Figure 2). At present, no available regulation on the permissible Mn content in fishery seafood exists.

Cu usually exists under the form of organic complexes and is distributed in the tissues of some human organisms. However, long-time exposure to Cu can affect human health such as liver and kidney damage [30]. The UK Food Standards Committee suggested that Cu concentrations in food should not exceed a value of 10.0 mg·kg^−1^ as wet weight [27]. In this study, Cu contents averaged 1.84 ± 1.13 mg·kg^−1^ for fish, 4.08 ± 2.22 mg·kg^−1^ for crustacean, and 5.52 ± 4.19 mg·kg^−1^ for mollusc samples (Table 4, Figure 2). The Cu mean value in fish samples was still far below the threshold of FAO/WHO [26]. For crustaceans, even though the maximal observed value (7.22 mg·kg^−1^) exceeded the EU threshold (5 mg·kg^−1^) [27], the average value in crustaceans was lower than the EU threshold (Table 4). No permissible value for Cu in molluscs for both national and international regulations is proposed.

Zn is an essential micronutrient to maintain certain biological functions for animals and humans. High Zn concentration however can lead to the loss of appetite, growth retardation, skin changes, comatose, and immunological abnormalities [31, 32]. In this study, the mean Zn contents were 17.51 ± 7.7 mg·kg^−1^ for fish, 20.61 ± 7.38 mg·kg^−1^ for crustaceans, and 25.82 ± 20.60 mg·kg^−1^ for mollusc samples (Table 4, Figure 2). The mean value of Zn contents in fish in this study was below the threshold of FAO/WHO (1989) [26]. For crustacean samples, the mean Zn value was below the EU 2006 threshold. No permissible Zn content in molluscs is proposed in both national and international regulations. However, the maximal value (82.71 mg·kg^−1^) was detected in *Meretrix* sp.

Different As species may be found in food, but organic As is less toxic than inorganic As (arsenite and arsenate) for human health. There is an elevated risk of cancer and other diseases or even death if high inorganic As is present in foods consumed. Lower As exposure can cause nausea and vomiting, decreased red and white blood cell production, and abnormal heart rhythms [32]. In this study, the total As concentrations averaged 1.10 ± 0.46 mg·kg^−1^ for fish, 2.40 ± 1.41 mg·kg^−1^ for the crustacean, and 1.99 ± 0.69 mg·kg^−1^ for mollusc samples. It is worth to noting that inorganic As was not analyzed in this study; thus, the results could not be compared to the permitted value of the Vietnam Ministry of Health QD 46–2007 BYT (2 mg·kg^−1^ for inorganic As). For crustaceans, As mean content was still lower than the Bangladesh (5 mg·kg^−1^) [33] and the EU seafood regulations. The As contents in mollusc samples in this study were close to that reported recently for the *Meretrix* sp. samples (1.284–2.553 mg·kg^−1^) cultured in the Thai Binh coastal zone [13].

Cd is a nonessential element for most living organisms. Cd can cause kidney failure, soften bones, and even cause prostate cancer due to long-term exposure or high doses [26, 32, 34]. In this study, Cd content averaged 0.02 ± 0.02 mg·kg^−1^ for fish, 0.26 ± 0.32 mg·kg^−1^ for crustaceans, and 0.25 ± 0.24 mg·kg^−1^ for mollusc samples (Table 4, Figure 2). The mean values of Cd contents in fish were below the threshold specified by the Vietnamese regulations of QCVN 8-2: 2011/BYT and QD 46-2007 BYT and by the FAO/WHO 1989 [26] or the European Community legislation [34] (Table 4). For crustaceans, note that the Cd content in the *Stomatopoda* sp. sample (1.11 mg·kg^−1^) was two folds higher than the Vietnamese threshold; however, the mean Cd value of all observed crustacean samples was below the threshold specified by the QCVN 8-2: 2011/BYT and QD 46-2007 BYT as well as the EU 2006 and by Bangladesh (0.5 mg·kg^−1^) [33].

Hg is a toxic element due to its strong affinity with the sulphur atom in the enzyme protein structure of living organisms. High Hg accumulation in human organisms can cause kidney failures, memory loss, numbness, physical tremors and neurological dysfunction, or other diseases (e.g., Minamata) [13]. In this study, Hg content averaged 0.19 ± 0.17 mg·kg^−1^ for fish, 0.40 ± 0.26 mg·kg^−1^ for crustaceans, and 0.22 ± 0.31 mg·kg^−1^ for mollusc samples. The mean Hg values in the fish, crustacean, and mollusc samples were lower than the threshold specified by the Vietnamese regulations of QCVN 8-2: 2011/BYT and QD 46–2007 BYT as well as other regulations (the EU 2006; Bangladesh's one) (Table 4). The Hg contents in mollusc samples in this study were close to those recently reported for the *Meretrix* sp. samples (0.045–0.472 mg·kg^−1^, average of 0.248 mg·kg^−1^) cultured in the Thai Binh coastal zone [13].

Our observation results showed that the trace metal element concentrations in fish, crustacean, and mollusc samples were in decreasing order: Fe > Zn > Mn > Cu > As > Hg ∼ Cd. For all samples observed, the mean values of Fe contents were always the highest, and Cd and Hg contents were the lowest.

### 3.2. Relationship between Trace Metal Element Accumulation in Fishery and Aquaculture Seafood

#### 3.2.1. Pearson and PCA Analysis

The results of the Pearson correlation coefficients for 7 trace metal elements of 35 observed samples showed that the significant relationship was found between Mn-Fe; Cu-Zn; Cd-As; and Hg-Cd (Table 5).

**Figure 3:**
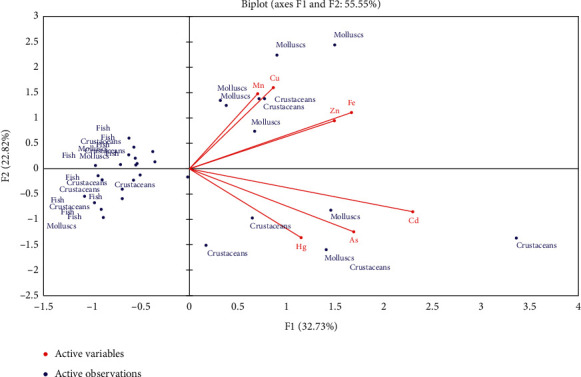
PCA analysis of 7 trace metal elements in 35 observed samples in the coastal zone of the Red River in Thai Binh and Nam Dinh provinces in 2020.

Relation between trace metal elements and biological groups is shown using PCA (Figure 3). The first two axes account for 55.5% of the variance, and the three conditions are clearly individualized: high level of Hg, As, and Cd accumulated in crustaceans samples is located in the right lower quarter; high level of Mn, Zn, Cu, and Fe accumulated in mollusc samples is located in the right superior quarter, and low metal accumulated in fish is located in the left quarter.

Organisms living in the aquatic environment are more likely to be exposed to trace metal elements, especially for benthic species. Crustaceans and molluscs are known to ingest or filter sediment; thus, metal accumulation in their bodies could be high [35]. Many studies have shown the high correlation between trace metal contents in sediment and in benthic organisms. Nour [7] studied on the contents of Fe, Mn, Cu, Zn, Pb, Ni, Cd, and Co in molluscan shells and associated surface sediments on the Gulf of Aqaba and Red Sea coasts, Egypt, and found that metal bioaccumulation of molluscan species was consistent with the enrichment factors for sediments. In another study, significant correlation of Cd, Cu, Pb, and Zn in sediments and oysters (*Saccostrea cucullata*) collected from Qeshm Island, Persian Gulf, Iran, was also demonstrated [36]. The authors revealed the soft tissues of *S. cucullata* as a more accurate biomonitoring organism for Cu, Pb, and Cd in sediments in this region [36]. Our results also showed that higher trace metal element contents in both crustaceans and molluscs in benthic sediment than in pelagic fishes (*p* < 0.05) were observed in the coastal zone of the Red River (Table 4, Figure 2). Moreover, in our study, some higher contents of trace metal elements (Cu, Cd, and Hg) than the allowed values for seafood safety were found for several crustaceans and mollusc samples, including *Meretrix* sp. High contents of trace metal elements (Cd and Hg) in *Meretrix* sp. in some estuaries of the central Vietnam have also been previously reported [37]. This indicates that the characteristic of filter feeding was one of the factors influencing higher accumulation rates and underlines why molluscs are widely used for water biomonitoring in coastal regions.

### 3.3. Factors Impacting on Trace Metal Element Accumulation in Fishery and Aquaculture Seafood

Gammal et al. [38] revealed that the mean contents of metals in crustaceans (shrimp and crab) were relatively higher than that in sediments, which may be related to some factors such as environmental quality, feeding strategies, metabolic activities, or dietary uptake of habitat. In comparison to other work, most trace metal element contents in seafood in the Red River coastal zone were much lower than that in Isobbe, Limbe, Cameroon [39], but far higher than that in Arabian Gulf, Saudi Arabia [38]. However, they were in the range as those from the North East Coast of India [40] or in Alexandria region, Egypt [41] (Table 6).

Previous studies also revealed that some trace metal element contents in coastal waters and sediments in this region were higher than in other coastal regions of Vietnam [12, 13, 21]. Duong et al. [21] reported that some trace metal elements (Cu, Hg, and Cd) in both water and sediment at Ba Lat site during the period from 2016 to 2019 were about 2-3 folds higher than at other sites along the coastal zone in North Vietnam, even though the mean values of all observed trace metal elements were lower than the allowed values defined by the Vietnamese regulations. Other studies demonstrated the high level of some trace metal elements, e.g., Fe and Zn, in the coastal water of the Red River in Thai Binh and Nam Dinh provinces [12–14]. Moreover, Hoai et al. [15] reported that the contents of some trace metal elements (mg·kg^−1^) in tidal flat sediment in the North Vietnam coast were as follows: Cu, 0.69–94.76 (mean: 40.50); Pb, 5.78–120.32 (mean: 52.08); Zn, 3.95–492.01 (mean: 85.31); As, 0.26–53.93 (mean: 18.73); Cd, 0.02–2.56 (mean: 0.44). These authors emphasized the high contents of some trace metal elements (Cu, Pb, As, and Cd), from moderately to strongly polluted environments in the Ba Lat water coastal zone and noted that the contamination increased with time. The higher trace metal element concentrations in both water and sediment may affect the bioaccumulation of the elements by benthic species in this region.

The difference in trace metal element contents in seafood may reflect the water and sediment quality which are impacted by both point and dispersal wastes sources. In our case, the pollution in the coastal water and sediment of the Thai Binh and Nam Dinh provinces probably comes from inland (domestic, agricultural, and industrial wastewater) [12, 15, 18]. Nguyen et al. [17] studied on trace metal element concentration in surface soil samples collected from different land use types in agricultural land in Nam Dinh Province and found that Cr mainly originated from a natural source. Cd and As have a significant anthropogenic input whereas Cd, Pb, and Zn have a mixed source. These authors suggested that Cd and As contamination in agricultural soil was possibly caused by sewage sludge, industrial wastewater, and/or residues of fertilizers and pesticides application. Phan [54] revealed the Cu contamination in soils of the industrial, agricultural zones, and craft villages in Thai Binh Province. Some authors [13, 16] reported high ranges in Hg content (from 0.12 to 3.79 mg·kg^−1^, averaging 0.98 mg·kg^−1^) in the surface sediment of the Red River coastal zone, probably due to the misuse of a remarkable amount of mercurial fungicides in large aquaculture production in Thai Binh Province. In addition, Nguyen et al. [55] reported that high As and trace metal element contents were observed in river sediment and in the upper soil layers of the mangrove forest soil of the Red River estuary which may reflect the intensive human activities in the upstream Red River in recent decades. Besides, Le Thi Lai et al. [11] reported that water channels in handicraft villages in Nam Dinh Province are loaded with trace metal elements (Zn, Pb, Cu, Cd, Cr, and Fe), exceeding the limits by up to 50 times. Thus, pollution sources need to be managed to ensure fishery and aquacultural seafood quality and safety, as well as for providing better environmental quality for the sustainable development of aquaculture in the Red River coastal zone.

In the Vietnam National technical regulation on the limits of heavy metal contamination in food (QCVN 8-2:2011/BYT), the allowed values of some trace metal elements such as arsenic, lead, cadmium, Mercury, and tin in seafood have been set. However, there is no regulation for other metals such as copper, manganese, zinc, and iron. The effects of these metals on human health have been shown in some studies [56–58]. However, risk assessments to human health of trace metal element accumulation in seafood in Vietnam are still limited. Thus, our results suggest that further studies of the risk assessment of trace metal elements in this study area and in all provinces of Vietnam are needed.

Our results on trace metal elements in fishery and aquaculture seafood are close to those reported in previous studies from the coastal zone in Vietnam such as in Cau Hai Lagoon [42], in Thai Binh Province [13], or in the North [21]. Similar levels of trace metal element contents in seafood in North Vietnam's coastal zone by using the green sample preparation and modern analytical equipment ICP-MS in this study and by other research methods (e.g., AAS analysis) was found. The ICP-MS method is more sensitive (detection at ppb levels) and can analyze multiple elements simultaneously as compared to the AAS analysis method which is cheaper but can only determine the concentration of a particular element. Moreover, our method using green sample preparation has its advantages for sample treatment as demonstrated above (Section 2.3). Thus, the method using the green sample preparation and an ICP-MS in this study should be applied for similar studies.

### 3.4. Limitation of This Study

Our study was conducted on limited variables (seven trace metal elements: Fe, Zn, Mn, As, Cu, Cd, and Hg) and sample size (number of samples); thus, these results of trace metal elements in seafood of the Red River coastal zone should be interpreted as preliminary. Besides, environmental (water and sediment) quality which may explain the accumulation of trace metal elements was not observed directly in this research. Thus, further investigation about the environmental and seafood quality should be undertaken for this region. In addition, our study only focused on total trace metal element contents which may not provide enough information about the biological activity and eco-toxicity; thus, further studies concerning a separate analysis of organic and inorganic forms of observed trace metal elements or the assessment of the potential risk of adverse health effects from a mixture of toxic metals are needed.

## 4. Conclusions

Using the green sample preparation and inductively coupled plasma-mass spectrometry (ICP-MS), different trace metal element (Fe, Zn, Mn, As, Cu, Cd, and Hg) contents in 35 samples (fish, crustacean and mollusc) in the coastal zone of the Red River in the Thai Binh and Nam Dinh provinces in four sampling campaigns in 2020 were analyzed. The results showed that the trace metal element contents in fish, crustacean, and mollusc samples were in the decreasing order: Fe > Zn > Mn > Cu > As > Hg ∼ Cd. In more details, the trace metal element contents (mg·kg^−1^) in all 35 samples varied from 0.48 to 22.73 for Mn; 13.13 to 202.73 for Fe; 0.72 to 15.58 for Cu; 7.63 to 82.71 for Zn; 0.18 to 5.12 for As; 0.001 to 1.114 for Cd; and 0.001 to 0.923 for Hg. Concentrations in fish samples were lower than in crustaceans and mollusc, showing the importance of sediment sources in providing heavy metal bioaccumulation in benthic organisms (crustaceans and mollusc).

Our research results provide a dataset for both fishery and aquacultural seafood qualities in the Red River coastal zone in Nam Dinh and Thai Binh provinces. Although the mean values of the different trace metal elements observed in this study were lower than the allowed values of Vietnam or the European threshold for seafood safety, some high concentrations were detected. Thus, the expansion of monitoring scope (increasing frequencies of sampling campaigns, numbers of samples, and numbers of variables of food quality) is required for obtaining a fully comprehensive assessment of seafood quality in this region. Our study only focused on total trace metal element contents which may not always provide enough information about biological activity and eco-toxicity. Thus, a separate analysis of organic and inorganic forms of observed trace metal elements is needed for accurately assessing the risks of toxic metals in seafood. In addition, our results also indicate the need for managing water and sediment quality in coastal areas, especially where aquaculture activities are carried out.

## Figures and Tables

**Table 1:** Sampling sites and sample information.

Types	Species	Number of samples	Date of sampling	Sample location
Fishes (13)	*Muraenesox cinereus*	1	Mar. 2020	Natural coastal zone
	*Pseudapocryptes elongates*	1	Jan. 2020	Natural coastal zone
	*Apogon sp.*	2	Jan. and Mar. 2020	Natural coastal zone
	*Trichiurus lepturus*	1	Jan. 2020	Natural coastal zone
	*Harpadon neheureus*	1	Jan. 2020	Natural coastal zone
	*Megalaspis cordyla*	1	Jan. 2020	Natural coastal zone
	*Chelon subviridis*	1	Jan. 2020	Natural coastal zone
	*Gobius sp.*	4	Jan., Mar., Jul. and August 2020	Natural coastal zone
	*Rachycentron sp.*	1	Jan. 2020	Natural coastal zone

Crustaceans (12)	*Metapenaeus sp.*	1	Mar. 2020	Natural coastal zone
	*Scylla sp.*	2	Jan. and Mar 2020	Natural coastal zone
	*Portunus sp.*	1	Jan. 2020	Natural coastal zone
	*Fenneropenaeus sp.*	1	Jan. 2020	Natural coastal zone
	*Penaeus sp.*	3	Jan., Jul. 2020	Natural coastal zone
	*Stomatopoda sp.*	1	Jan., Mar. 2020	Natural coastal zone
	*Donax sp.*	2	Mar. and Jul. 2020	Natural coastal zone
	*Mierspenaeopsis hardwickii*	1	Jul. 2020	Natural coastal zone

Molluscs (10)	*Sepiola sp.*	1	Jul. 2020	Natural coastal zone
	*Loliginidae sp.*	1	Jul. 2020	Natural coastal zone
	*Octopus sp.*	1	Jul. 2020	Natural coastal zone
	*Anadara sp.*	1	Mar. 2020	Natural coastal zone
	*Crassostrea sp.*	1	Jan. 2020	Natural coastal zone
	*Meretrix meretrix, Meretrix lyrata, Meretrix sp.*	5	Jan., Mar., Jul., and Aug. 2020	Aquaculture coastal zone

Total samples	35		

**Table 2:** Operating parameters of the ICP-MS system.

Parameters	Value
RF power	1250 W
RF matching	1.45 V
Sample delay	90 s
Sample uptake flow	0.1 ml·min^−1^
Sample depth	6.4 mm
Plasma gas	15 L·min^−1^
Carrier gas flow	1.2 L·min^−1^
Auxiliary gas flow	0.9 L·min^−1^
Pressure analytical	3.10^−4^–2.10^−3^ Pa
The ion lens voltage	5.75 V
Coolant flow	2.2 L·min^−1^
Coolant temp	2°C
Data acquisition:	
Peak pattern	Full quant (3)
Integrations time	0.1 s
Repetition	3
Analytical mass	Fe (57), Zn (66), Mn (55), As (75), Cu (63), Cd (111), Hg (202)

**Table 3:** LOD and LOQ values of analyzed trace metal elements by ICP-MS.

Element	*S*	Blank signals *I*_blank_	Standard signals *I*_stand_	Standards concentration (*μ*g·L^−1^)	LOD (*μ*g·L^−1^)	LOQ (*μ*g·L^−1^)
Cu	1.37	5.92	597.36	10	0.07	0.23
Cd	1.27	4.51	3963.70	10	0.01	0.03
Zn	1.70	6.75	50.35	10	1.17	3.90
Fe	3.79	255.26	1012.41	10	0.15	0.50
Mn	1.55	175.75	3359.39	10	0.02	0.05
As	1.42	2.38	453.91	10	0.09	0.31
Hg	1.43	4.42	165.33	10	0.27	0.89

**Table 4:** Average (min-max) values of heavy metal contents (mg·kg^−1^) in fish, crustacean, and mollusc samples.

Samples		Mn	Fe	Cu	Zn	As	Cd	Hg
Fish	Average	**5.99**	**51.23**	**1.84**	**17.51**	**1.10**	**0.02**	**0.19**
	Min-max	1.46–17.06	38.02–76.54	0.72–3.89	7.63–31.26	0.18–1.76	0.001–0.076	0.01–0.54
	QCVN 8-2:2011/BYT	—	—	—	—	—	*0.1*	*1*
	QĐ 46-2007 BYT	—	—	*30*	*100*	*2* ^*∗*^	*0.05*	*0.5*
	FAO/WHO 1989 [26]	—	—	*30*	*40*	—	*0.5*	—

Crustacean	Average	**6.37**	**89.92**	**4.08**	**20.61**	**2.40**	**0.26**	**0.40**
	Min-max	0.78–19.90	31.15–195.76	1.10–7.22	12.68–40.35	0.87–5.12	0.04–1.11	0.001–0.923
	QCVN 8-2:2011/BYT	—	—	—	—	—	*0.5*	*0.5*
	QĐ 46-2007 BYT	—	—	—	—	*2.00* ^*∗*^	*0.5*	*0.5*
	EU 2006 [27]	—	—	*5*	*50*	*5*	*0.5*	*0.5*

Mollusc	Average	**8.24**	**113.14**	**5.52**	**25.82**	**1.99**	**0.25**	**0.22**
	Min-max	0.48–22.73	13.13–202.73	2.12–15.58	9.75–82.71	1.06–3.32	0.004–0.85	0.01–0.79
	QCVN 8-2:2011/BYT	—	—	—	—		*0.5*	*0.5*
	QĐ 46-2007 BYT	—	—	—	—	*2.00*	*0.5*	*0.5*

*Note.* ^*∗*^Inorganic As concentration in seafood. Bold values indicate that they are the mean (average) values for 7 trace metal elements in three kinds of seafood.

**Table 5:** Correlation between seven trace metal elements of 35 observed samples.

	Mn	Fe	Cu	Zn	As	Cd	Hg
Mn	1						
Fe	**0.638 ** ^*∗∗*^	1					
Cu	−0.017	0.208	1				
Zn	0.021	0.113	**0.596 ** ^*∗∗*^	1			
As	−0.131	0.256	−0.105	0.159	1		
Cd	0.043	0.368^*∗*^	0.074	0.404^*∗*^	**0.639 ** ^*∗∗*^	1	
Hg	−0.043	0.064	−0.093	0.003	0.203	**0.498 ** ^*∗∗*^	1

*Note*. Values in bold letters show significant correlations ^*∗*^Correlation is significant at the 0.05 level (2-tailed) ^*∗∗*^Correlation is significant at the 0.01 level (2-tailed).

**Table 6:** Trace metal elements in some aquatic species in estuary/coastal zones in the world.

	Location	Aquatic species	Mn (mg·kg^−1^)	Fe (mg·kg^−1^)	Cu (mg·kg^−1^)	Zn (mg·kg^−1^)	As (mg·kg^−1^)	Cd (mg·kg^−1^)	Hg (mg·kg^−1^)	References
Fish	Cau Hai Lagoon, Vietnam		5.2	39	0.8	58	2.1	0.0074	—	[42]
	Karnaphuli River estuary, Bangladesh		—	—	12.1 (10.27–16.41)	—	4.89 (3.19–6.19)	0.39 (0.21–0.74)	—	[43]
	North East Coast of India	Fish	0.5–12.0	10.4–249.7	0.5–28.2	3.0–99.1	0.02–2.37	0.01–1.10	0.05–1.60	[40]
	Karachi Fish Harbour, Pakistan		—	—	—	—	—	—	0.042 ± 0.023 (0.01–0.09)	[44]
	Arabian Gulf, Saudi Arabia		—	—	—	—	0.11–0.61	—	0.11–1.24	[45]
	Red River coastal zone, Vietnam		5.99	51.23	1.84	17.51	1.10	0.02	0.19	This study

Crustacean	Lagos Lagoon, Badore, Nigeria	Crab	11.941 ± 1.538	—	—	6.389 ± 0.905	—	0.009 ± 0.001	0.004 ± 0.001	[46]
	Gresik coast, Indonesia	Shrimp (in 2005) and (in 2004–2008)	—	—	1.166 ± 0.162	2.132 ± 0.280	0.084 ± 0.015	0.0002 ± 0.0001	<0.0006–0.0082	[47, 48]
	Alexandria coastal zone, Egypt	Crab, shrimp (prawn)	—	9.2 (6.6–11)	3.25 (1.4–6.4)	22 (19–27)	—	0.074 (0.04–1.47)	0.15 (0.07–0.29)	[41]
	Isobbe, Limbe, Cameroon	Crab	51.13	332.49	101.16	42.09	—	0.067		[39]
	Arabian Gulf, Saudi Arabia	Shrimp	—	0.085	Not detect	29.521	25.527 or 0.19–0.53	—	0.0512 or 0.13–0.91	[38] or [45]
		Crabs	—	0.075	Not detect	31.984	20.666	—	0.0580	[38]
	Red River coastal zone, Vietnam	Crustacean	6.37	89.92	4.08	20.61	2.40	0.26	0.40	This study

Mollusc	Arabian Gulf, Saudi Arabia	Squids	—	0.068	Not detect	63.396	30.069	—	0.1065	[38]
	Alexandria coastal zone, Egypt	Bivalves (*Mactra* sp.; *Mytilus* sp.)	—	9.9 (7.2–18.7)	1.15 (0.5–2.5)	20 (17–35)	—	0.054 (0.03–0.19)	0.135 (0.01–0.33)	[41]
	Seijang estuary, Indonesia	Mollusc (*Geloina* sp. and *Calliostoma* sp.)	—	—	3.45–34.29	20.28–56.54	—	—	—	[49]
	Bintan Island, Indonesia	Bivalves	—	—	—	—	—		0.01–0.26	[50]
	Bohai Sea, China	Bivalves, gastropods	—	—	1.16–172.25	9.95–705		0.14–30.61	0.027–0.46 [51]	[51, 52]
	Sarawak river estuary	Bivalves (*Polymesoda expansa*, *Meretrix meretrix*, and *Solen regularis*)	—	177.82–295.31	0.84–2.21	24.13–62.24	—	1.15–2.35	—	[53]
	Coastal zone, Thai Binh Province, Vietnam	Clam (*Meretrix* sp.)	—	—	—	—	1.80 (1.26–2.55)	—	0.25 (0.05–0.47)	[13]
	Coastal zone in North Vietnam	Clam (*Meretrix* sp.)	0.14–3.06	34.88–752.31	2.74–35.15	16.66–211.13	0.13–0.41	0.09–2.00	0.03–0.14	[21]
	Red River coastal zone, Vietnam	Mollusc (clam, oysters, squid octopus)	8.24	113.14	5.52	25.82	1.99	0.25	0.22	This study

## Data Availability

All the data and supporting materials are included within the article.
